# A Study on Drilling High-Strength CFRP Laminates: Frictional Heat and Cutting Temperature

**DOI:** 10.3390/ma11122366

**Published:** 2018-11-25

**Authors:** Jinyang Xu, Chao Li, Jiaqiang Dang, Mohamed El Mansori, Fei Ren

**Affiliations:** 1 School of Mechanical Engineering, Shanghai Jiao Tong University, Shanghai 200240, China; mrlilynn@sjtu.edu.cn (C.L.); jqdang@sjtu.edu.cn (J.D.); 2 MSMP—EA 7350, Arts et Métiers ParisTech, Châlons-en-Champagne 51006, France; mohamed.elmansori@ensam.eu; 3 Department of Mechanical Engineering, Texas A&M University, College Station, TX 77840, USA; 4 Shanghai Aerospace Equipments Manufacturer Co., Ltd., Shanghai 200245, China; renfei_149@163.com

**Keywords:** high-strength CFRP composites, drilling, frictional heat, cutting temperature

## Abstract

High-strength carbon fiber reinforced polymer (CFRP) composites have become popular materials to be utilized in the aerospace and automotive industries, due to their unique and superior mechanical properties. An understanding of cutting temperatures is rather important when dealing with high-strength CFRPs, since machining defects are likely to occur because of high temperatures (especially in the semi-closed drilling process). The friction behavior at the flank tool-workpiece interface when drilling CFRPs plays a vital role in the heat generation, which still remains poorly understood. The aim of this paper is to address the friction-induced heat based on two specially-designed tribometers to simulate different sliding velocities, similar to those occurring along the flank tool-work interface in drilling. The elastic recovery effect during the drilling process was considered during the tribo-drilling experiments. The drilling temperatures were calculated by the analytical model and verified by the in-situ experimental results gained using the embedded thermocouples into the drills. The results indicate that the magnitudes of the interfacial friction coefficients between the cemented carbide tool and the CFRP specimen are within the range between 0.135–0.168 under the examined conditions. Additionally, the friction caused by the plastic deformation and elastic recovery effects plays a dominant role when the sliding velocity increases. The findings in this paper point out the impact of the friction-induced heat and cutting parameters on the overall drilling temperature.

## 1. Introduction

High-strength carbon fiber reinforced polymer (CFRP) composites have become one of the most popular materials in many industries, including aerospace, automobile, etc., due to their excellent mechanical properties of high specific tensile strength, fracture toughness, modulus and fatigue strength. Meanwhile, the CFRP materials meet the lightweight requirements for aircraft structures in order to reduce fuel consumption in regard to economic and environmental reasons while maintaining durability and safety standards [1,2]. Generally, CFRP components are manufactured by prepregs utilizing carbon fibers impregnated with thermoset resin. Prepreg sheets are formed by laying up and heat curing. Even so, a variety of the cured CFRP parts still require a secondary machining to satisfy the final dimensional accuracy, since the layered prepreg sheets possess a redundant edge. Mechanical joining is also inevitable for the assembly process of CFRP parts. Compared with the joining of metals by welding, CFRP composites are often assembled via the use of bolt or rivet connections, requiring a large number of holes to be drilled in the structural components. Additionally, some composite parts need drilling operations to accommodate hardware and other fasteners [3].

Regarding the development of materials, as well as the requirement of high efficiency/quality in drilling process, a large number of novel methods are being employed for the drilling of high-strength CFRP, such as laser beam drilling [4,5], abrasive water jet drilling [6,7], rotary ultrasonic drilling [8] and electrical discharge drilling [9,10]. A number of investigations have been conducted with respect to the involved non-conventional methods. Laser beam drilling is one of the most important unconventional machining processes that is employed in machining the high-strength steels, metal alloys, ceramics, composites and superalloys with satisfaction. Abrasive water jet processing generally induces no thermal damage in the machined surface; however, the machining cost is relatively high. Additionally, it is of great difficulty of processing where the jet does not penetrate. Rotary ultrasonic drilling has been widely used in hole-drilling of brittle materials and composites owing to its advantage of reducing cutting forces and improving machining quality. The electrical discharge drilling has been proved to be a feasible technology in producing defect-free holes. Although the non-conventional hole-making technologies have been widely studied in detail, conventional drilling using the standard drill bits still remains one of the most economical operations for practical purposes [11].

It is more challenging to drill high-strength CFRP composites reinforced with long fibers compared with some metal materials. For instance, the presence of uncut polyester fibers often leads to an increase of machined surface roughness [12]. The chip removal mechanism of the CFRP composite is quite different from that of metals in view of the fact that CFRP composites are characterized by multi-scale characteristics in nature and anisotropy in properties [13]. Besides, resins are easier to fracture than fibers and the adhesive strength between resins and fibers are relatively lower compared with the fracture strength of the reinforcing fibers [14]. Hence, mechanical defects, such as tearing, spalling, delamination and fiber pullout are more likely to occur during the drilling process [15]. To address the aforementioned machining defects, a large number of analytical and numerical models have been developed by achieving some favorable results. However, to obtain drilled holes with high quality the machinists should not only deal with the mechanical damage induced in drilling, but also pay more attention to the issue of the cutting temperature caused by the interaction between the drill bit and the workpiece, since it greatly affects the machined surface quality and the tool life. For a typical CFRP material, the glass transition temperature is approximately 180 °C, over which resin degradation occurs within the subsurface layers. The resin degradation is more likely to induce interlaminar delamination, fiber/matrix interface debonding and reduce the strength of the material, exacerbating the extents of the machining defects [16].

A variety of temperature measuring methods utilized to monitor different machining processes, combining analytical, numerical and experimental techniques, were performed in the open literature. The method of embedding thermocouples into the drill bit remains the most-used and the earliest technique to capture the cutting temperatures and thermal gradients of the cutting edges during the drilling process. Samy et al. [17] analyzed the effects of cutting parameters on the temperature, thrust force and surface roughness in drilling composites where the methods of embedded thermocouples and infrared camera were used. The results indicate that a larger point angle at higher spindle speeds increases the temperature developed in the surface of holes, which is attributed to the high frictional contact between the tool and the workpiece. Brinksmeier et al. [18] made a comparative study between the conventional drilling and orbital drilling of aluminum/CFRP/titanium composites in terms of the cutting temperature measured by the embedded thermocouples into the tool tip. The authors reported that the orbital drilling induced lower cutting temperatures compared with the conventional drilling under the conditions of identical cutting speeds and the surface integrity of boreholes produced by the orbital drilling is much better than the conventional drilling. Another method is the use of wireless systems to transmit the acquired data from the spindle during the machining of composites [19,20]. Additionally, a number of non-contact techniques, such as infrared radiation were utilized to measure the temperatures developed in different machining operations [21,22]. Apart from the above studies, the thermal behavior and heat transfer mechanisms of fibrous composites were studied by several researchers. Tian and Cole [23] studied the thermal properties of CFRP composites and concluded that the fibrous composite presented an anisotropic thermal conductivity varying in the in-plane and through-thickness directions. Merino-Pérez et al. [24] studied the influence of material properties and cutting speeds on the heat dissipation during drilling CFRP by adopting both the methods of the embedded thermocouples and the infrared camera. The results implied that the cross-linking density of the matrix, the crystallinity, as well as the structure of the reinforcing fibers affect the heat dissipation and the overall temperatures.

However, issues relating to the heat generation in the material removal when drilling the fibrous composites are still not widely addressed in the literature. Regarding the conventional drilling process, the input energy is generally dissipated by two parts, namely the chip separation and the friction at the tool-chip and tool-work interfaces. Since the CFRP chips are separated by brittle fracture without undergoing elastoplastic deformations, the heat produced by the chip breakage is relatively low, which can be reasonably neglected. In contrast, a large amount of the cutting heat is dissipated by the chips. The powder-like composite chips would reduce the contact length of the tool-chip interaction zone and further suppress the frictional heat generation at the tool-chip interface. However, the interaction at the tool-work interface plays a vital role in the frictional heat generation because of the elastic recovery effects. Additionally, the friction-induced heat can easily accumulate at the tool flank face, which may dissipate into the machined CFRP surface particularly in the semi-closed drilling operation. The present study is an extension of our previous work [25] aiming to eliminate the errors induced by the method of designing experiments, which will be discussed in detail in the following subsections and to address fundamentally the friction-induced heat in drilling CFRP composites. The drilling temperature and friction-induced temperature were recorded in-situ through the use of the thermocouples embedded into the drills and pins, respectively. The obtained results were correlated to the used cutting parameters.

## 2. Materials and Methods

### 2.1. Workpiece Specimens

**Figure 1:**
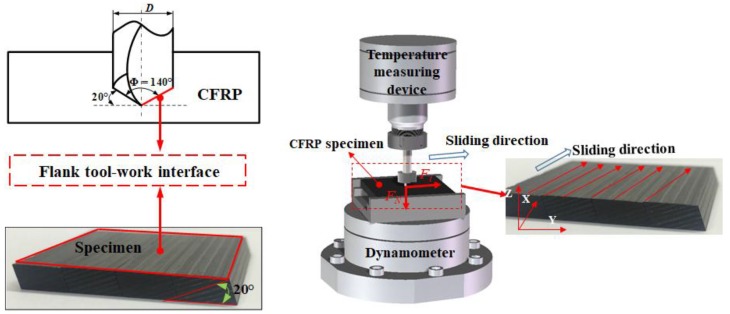
The specially-designed carbon fiber reinforced polymer (CFRP) plate for replicating the local friction conditions of drilling and the scheme of the pin-on-disc tests.

The composite plates investigated in this work were multilayer high-strength CFRP laminates fabricated by carbon fibers and epoxy prepregs containing a 65% fiber volume fraction. The matrix is an X850 epoxy material and the reinforcement is T800 carbon fibers. The physical properties of the composite specimens are given in Table 1. Two different structures of CFRP specimens were adopted in the present work consisting of a standard laminate for the drilling tests and a modified laminate for the pin-on-disc tests that was milled to expose the contact surface according to the tool geometry, analogous to the flank tool-work interface between the cutting edge and the workpiece. The plate was cut off to the size of 45 mm × 40 mm × 5 mm, the structure of which is depicted in Figure 1.

### 2.2. Experimental Setup

**Figure 2:**
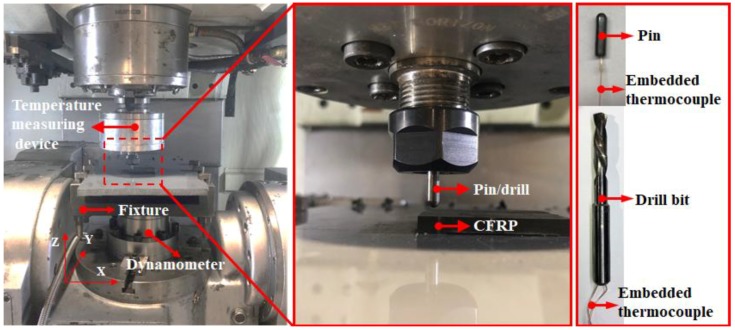
The experimental setup for the pin-on-disc tests and the used pin/twist drill.

The HURCO VMX42 vertical machining center (Hurco, Indianapolis, IN, USA) as used for the drilling and pin-on-disc experiments, as shown in Figure 2, which has a maximum rapid feed rate of 30 m/min and a positioning accuracy of 0.01 mm. A 6.35-mm diameter uncoated carbide twist drill (SD203A-02500-091-0315R1-T) with two inner coolant holes provided by the Seco Tools (Fagersta, Sweden) was utilized, as shown in Figure 2. The drill bit was characterized by a point angle of 140°, a helix angle of 35°, two cutting edges and a chisel edge of 0.12 mm. During the drilling and pin-on-disc tests, the CFRP specimen was clamped by a fixture mounted on a piezoelectric dynamometer (Kistler 9272, Winterthur, Switzerland). Then, the recorded signals were amplified and collected via a multichannel charge amplifier (Kistler 5070A, Winterthur, Switzerland) and a data acquisition system, respectively, at an acquisition frequency of 20 kHz. All the experiments were conducted without coolants (dry conditions).

### 2.3. Temperature Measurement

**Figure 3:**
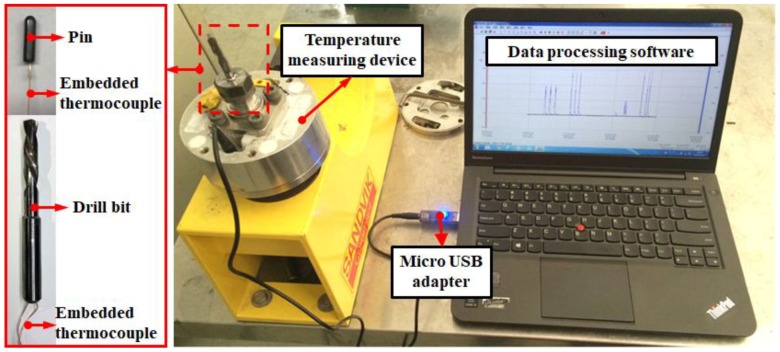
Photographs showing the setup of the temperature measuring system.

The thermocouples were embedded into the drill bits and the pins as illustrated in Figure 2 to measure the cutting temperature at the tool flank face and frictional heat produced by the interaction of the pin and CFRP specimen, respectively. This investigation utilized the insulated K-type thermocouples supplied by OMEGA Engineering, which have a working temperature ranging from −200 °C to +1370 °C and a length of 15 cm. The measuring accuracy of the temperature system is ±0.1 °C. According to the specifications given by the manufacturer, the used thermocouples are characterized by a response time of 0.22 s that is defined as the required time for the thermocouples to acquire 63.2% of the final temperature when the bead is alternately exposed in two different temperatures [24]. During the drilling and tribological experiments, the temperature data were recorded using a micro temperature storage module, in which the temperature signals were not extracted in-situ. After the completion of the tests, the temperature data were transmitted to the computer through a micro USB adapter for the further post-process analysis. The experimental setup of the temperature measuring device is shown in Figure 3.

### 2.4. Experimental Details

#### 2.4.1. Drilling Tests

A number of drilling tests were arranged to figure out the correlations between the drilling temperatures and the cutting parameters. Through the literature survey, it is believed that low feed rates (0.01–0.05 mm/rev) are favorable for the drilling of fibrous composites [26]. Hence, four levels of low feed rates (*f* = 0.01, 0.02, 0.03, 0.04 mm/rev) were adopted in the present work. Meanwhile, the cutting speeds were carefully selected to meet the conditions of the pin-on-disc tests. Table 2 summarizes the used cutting parameters for the drilling of CFRP laminates. The thrust forces and the drilling temperatures were in-situ measured.

#### 2.4.2. Pin-on-Disc Tests

To quantitatively evaluate the friction-induced heat, two types of tribometers were designed which were designated as pin 1 and pin 2. Being as a standard cemented carbide tool, pin 1 was utilized to replicate the frictional heat generated in the tool-work interaction zone during the drilling of CFRP composites. Additionally, pin 2, which was removed one half of its head, was adopted to avoid the elastic recovery effects during the drilling of CFRP composites. It is worth noting that the contact surfaces of the two pins were polished to guarantee a low surface roughness (*R_a_* < 0.30 μm). Then, the surface condition of the pins is similar to that of a finely carbide cutting tool [27]. The main drawback of our previous work [25] is the reduction of the thrust force caused by the wear consumption of the specimen surface during the reciprocating circular motion of pins in the tribological tests. To eliminate the experimental errors of interfacial friction coefficients, the rotary movement of the pins was replaced by the rectilinear motion in the present study as depicted in Figure 1. Meanwhile, considering the limitation of the rapid feed rate of the machine tool in the X and Y directions, the maximum sliding velocity was set as 30 m/min.

**Figure 4:**
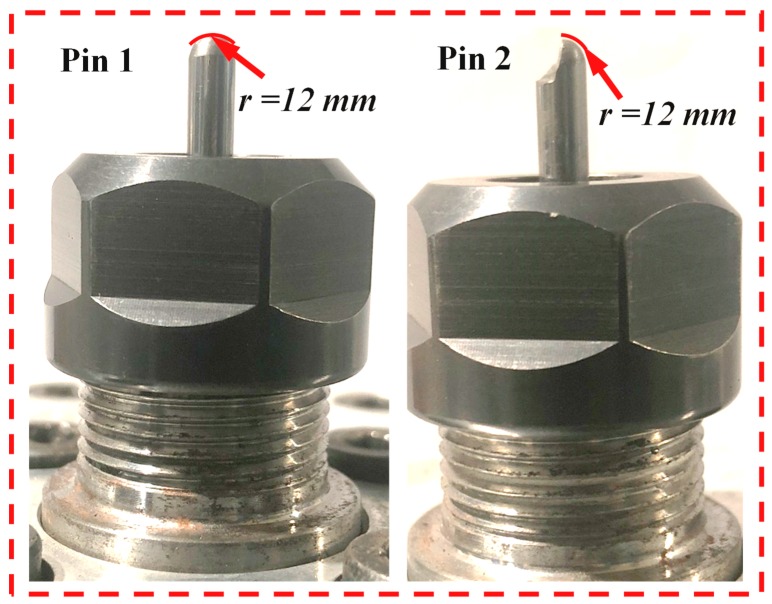
Photographs showing the two designed pins.

**Figure 5:**
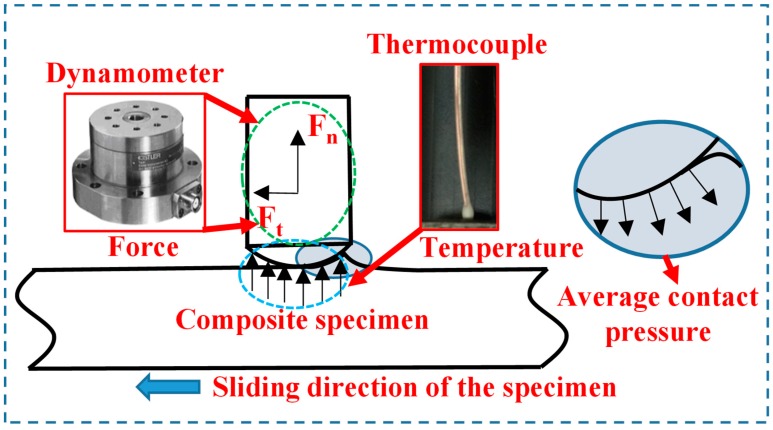
A schematic illustration of the working conditions for the tribometer [25].

In general, the pin-on-disc experiments were carried out following several steps to determine the interfacial friction coefficients while drilling the CFRP composites by referring to the work done by Mondelin et al. [28]. Firstly, the pin was mounted on the spindle of the CNC machine tool. Then, the pin moved vertically to touch the designed point of the CFRP specimen. In this step, the dynamometer signal was carefully observed to match the thrust force value obtained in the previous drilling tests. After reaching the set-point of the contact load, the pin stops to move. Then, a rectilinear movement in the Y direction under the configured rapid feed rate is applied to create a relative motion between the pin and the CFRP specimen that aims to replicate the contact conditions of the tool-work interface of drilling. The images showing the morphologies of two used pins are given in Figure 4. The CFRP specimen is fixed onto a dynamometer to measure the macroscopic normal load (*F_n_*) and tangential load (*F_t_*). The thermocouples were embedded into the pins to record the temperature during a test. A schematic diagram showing the working conditions of the tribometer is given in Figure 5.

### 2.5. Predictions of the Friction-induced Temperatures

#### 2.5.1. Modeling of Interfacial Friction Coefficients

The ratio of tangential force (*F_t_*) to normal force (*F_n_*) was defined as the interfacial friction coefficient (*μ_app_*), which signifies the macroscopic friction coefficient of the flank tool-work interface as expressed by the following equation.

(1) $$\;\mu_{app}=\frac{F_{t}}{F_{n}},\;$$

where *F_n_* denotes the thrust force in the Z-axis direction and *F_t_* represents the force in the X-axis direction obtained by the dynamometer.

The apparent friction coefficient (experimental value) was calculated based on the microscopic forces measured by the dynamometer. These forces include on the one hand the adhesive part influenced by material properties, such as hardness and chemical reactivity, and on the other hand the plastic deformation of the specimen, which cannot be ignored under the severe contact conditions [28,29]. The *μ_app_* consists of two parts: One is the adhesion part (*μ_adh_*) and another is the plastic deformation part (*μ_def_*). The elastic friction coefficient (*μ_ela_*) corresponding to the elastic recovery part was considered in this paper, due to the occurrence of the elastic recovery effects on the machined surfaces (Equation (2)).

(2) $$\mu_{app}=\mu_{adh}+\mu_{def}+\mu_{ela}.$$

Therefore, according to our previous work [25], the interfacial friction coefficient (*μ*) between the tool flank surface and the workpiece can be calculated by

(3) $$\mu =\mu_{adh}+\mu_{ela}.$$

#### 2.5.2. Analytical Predictions of the Friction-induced Heat

The prediction model of the frictional heat was established based on the Fourier’s law. The amount of heat transferred through a unit area per unit time is proportional to the temperature gradient, which can be expressed as follows:

(4) $$\;q=\frac{\Delta Q}{A\Delta t}=-\lambda \cdot \frac{dT}{d\delta },\;$$

where *q* is the heat flux density, ΔQ/Δt is the heat flow rate, A is the contact surface area, *λ* is the thermal conductivity coefficient, and dT/dδ is the temperature gradient.

During the machining of CFRPs, the tribological interaction between the tool flank surface and the machined composite surface plays a vital role in heat generation, due to the elastic recovery effects arising from the highly abrasive carbon fibers. To evaluate the temperature rise induced from the drilling operation, a prediction model was established based on the following assumptions with a reference to formulas describing the average temperature increment of the tool-chip interface:

- The frictional heat model was considered as a plane thermal source case.
- The average velocity of the main cutting edges was used in the model and the chisel edge length was neglected.
- The fracture-induced heat arising from two main fracture zones and the heat induced by the friction at the tool-chip contact surface were ignored.

A prediction model of the temperature rise for the composites drilling was derived from [30] as shown below:

(5) $$\Delta T=\frac{0.754R_{h}q_{h}}{k_{w}}\sqrt{\frac{a_{w}L_{w}}{V_{c}}},$$

where *R_h_* is the percentage of heat transferred to workpiece in this case, *q_h_* is the heat flux density of the flank-workpiece interaction zone, *k_w_* is the heat conductivity of work materials, *a_w_* is the thermal diffusivity of work materials, *L_w_* is the length of flank-workpiece interaction zone, and *V_c_* is the average cutting velocity of the drill lips.

(6) $$\;a_{w}=\frac{k_{w}}{\rho c_{w}},\;$$

where *ρ* and *c_w_* are the density and specific heat capacity of the composite, respectively. Assuming that all the work done by friction is transformed into heat, then

(7) $$\;q_{h}=\frac{\mu F_{N}V_{c}}{S_{flank}},\;$$

where *t* = *D*/(2sin(*φ*/2)) is the length of the drill cutting edge; *D* is the drill diameter; *φ* is the drill point angle; *F_n_* is the component of the thrust force that is perpendicular to the cutting plane. Taking the area that is cut across by the drill edges per revolution as the heat source plane, the following equation can be obtained:

(8) $$\;L_{w}t=\int_{0}^{l}2\pi \cdot l\cdot \sin\frac{\varphi }{2}dl,\;$$

Finally, the temperature localized at the CFRP drilling surface can be estimated by

(9) $$\;T=\frac{0.754R_{h}\mu F_{N}V_{c}\sin\frac{\varphi }{2}}{k_{w}L_{w}t}\sqrt{\frac{k_{w}L_{w}}{\rho c_{w}V_{c}}}+T_{r},\;$$

where the symbol *T_r_* denotes the room temperature.

## 3. Results and Discussion

### 3.1. Interfacial Friction Coefficient Assessment

**Figure 6:**
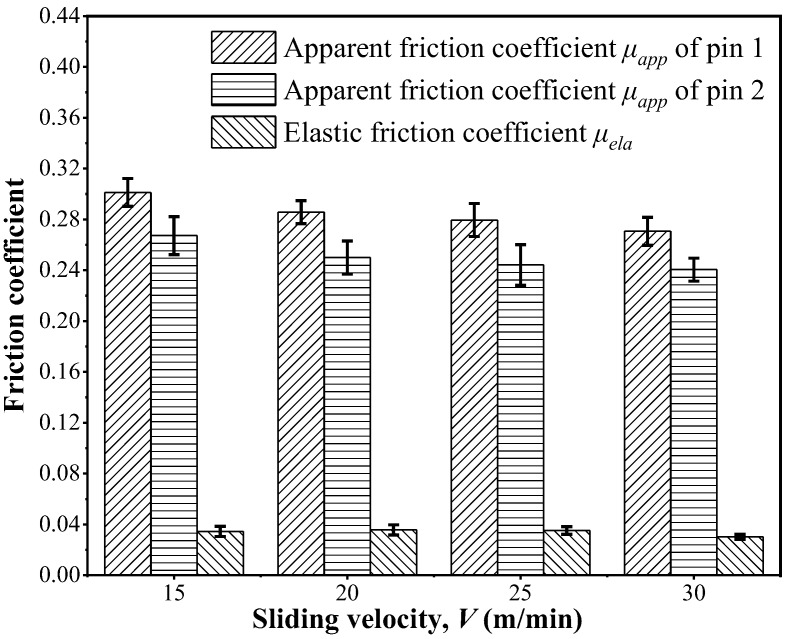
The apparent friction coefficients of the two pins and the elastic friction coefficients vs. the sliding velocity.

The correlations between the apparent friction coefficients (*μ_app_*) of two designed pins and the sliding velocity in the pin-on-disc tests, as well as the elastic friction coefficient (*μ_ela_*) calculated are given in Figure 6. It is clear that the magnitudes of the apparent friction coefficients for pin 1 are within the range of 0.27–0.31 under the examined conditions. Since pin 2 was removed one half of its head, the effects of the elastic recovery occurring at the tool-work interaction zone were totally eliminated. As such, magnitudes of the apparent friction coefficients of pin 2 are lower than those gained in the case of pin 1 as depicted in Figure 6. For a specific contact pressure, the apparent friction coefficients of two pins present a slightly decreasing trend as the sliding velocity increases. The findings were also reported in the research work done by Klinkova et al. [27] when investigating the friction properties between a randomly structured CFRP specimen and a TiN-coated carbide pin. Note that the *μ_app_* values obtained by Klinkova et al. [27] are lower than those developed in the present work under the identical sliding velocity range. This is attributed to the TiN coating of the pin in the work of Klinkova et al. [27] which greatly reduces the adhesion part of the apparent friction coefficient. Furthermore, it can be drawn that the magnitudes of the elastic friction coefficient (*μ_ela_*) were calculated around 0.04 with a very slight variation as the sliding velocity increases. Due to the elastic recovery effects between the machined surface and the tool flank surface, the elastic friction coefficient contributes to the increase of the frictional heat.

**Figure 7:**
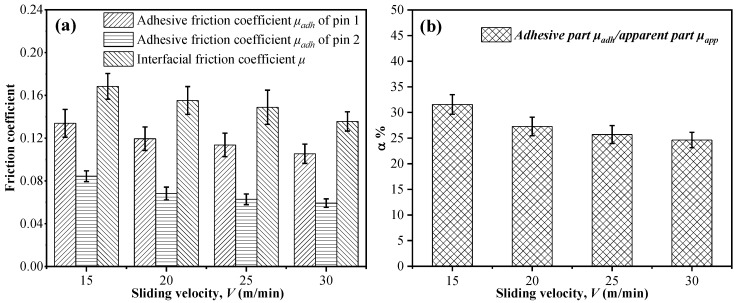
(**a**) The adhesive friction coefficients, the interfacial friction coefficients of the two pins and (**b**) the *μ_adh_*/*μ_app_* vs. the sliding velocity.

Evolutions of the adhesive friction coefficient, the interfacial friction coefficient of two pins and the *μ_adh_*_/_*μ_app_* vs. the sliding velocity are plotted in Figure 7. The downtrend of *μ_adh_*/*μ_app_* shows that the friction caused by the plastic deformation and elastic recovery effects plays a dominant role as the sliding velocity increases. This phenomenon is quite different from that encountered in machining metals. The adhesive friction coefficient basically does not change as the cutting velocity varies and usually plays a key role in the metal cutting. Furthermore, both the apparent friction coefficient (*μ_app_*) and adhesive friction coefficient (*μ_adh_*) are found to show a decreasing trend with the elevated sliding velocity. This is attributed to the thermal softening effects as the sliding velocity increases during the drilling of CFRPs. Moreover, the magnitudes of the frictional temperatures can be calculated according to Equation (9), which are discussed in the following subsection.

### 3.2. Analysis of the Friction-induced Heat

**Figure 8:**
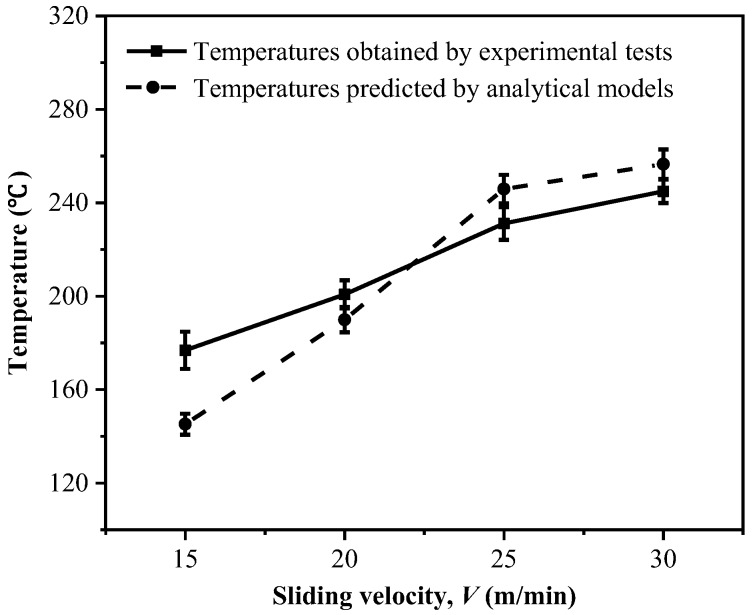
Comparison of the drilling temperatures of the tool-work interface gained by the experimental tests and analytical models at a constant feed rate (*f*) of 0.02 mm/rev.

Figure 8 shows a comparison of the temperatures at the tool-work interface zone gained by both experimental tests and analytical models. The results indicate that the temperatures developed at the tool-work interface show a high sensitivity to the sliding velocity and the temperature increases gradually as the sliding velocity is elevated. Meanwhile, the temperatures predicted by the analytical model are basically consistent with the results obtained by the experimental tests. Both the results indicate that higher temperatures are produced particularly under a higher sliding velocity. It is worth noting that the slight difference between the experimental results and analytical results is affected by some physical phenomena generated in the actual experimental process. The elevated temperature is detrimental to the drilling of CFRP composites, due to the increased risk of glass transition. Also, the high temperature promoted at the tool-work interface as the velocity increases would cause thermal softening of the composites, leading to matrix degradation. It could also consequently decrease the friction coefficients of *μ_app_* and *μ_adh_*, as discussed earlier in Section 3.1. Moreover, the results given in Figure 8 imply that a low cutting velocity is favorable for the drilling of high-strength composites in order to minimize the detrimental effects caused by the cutting temperature.

### 3.3. Drilling Temperature

**Figure 9:**
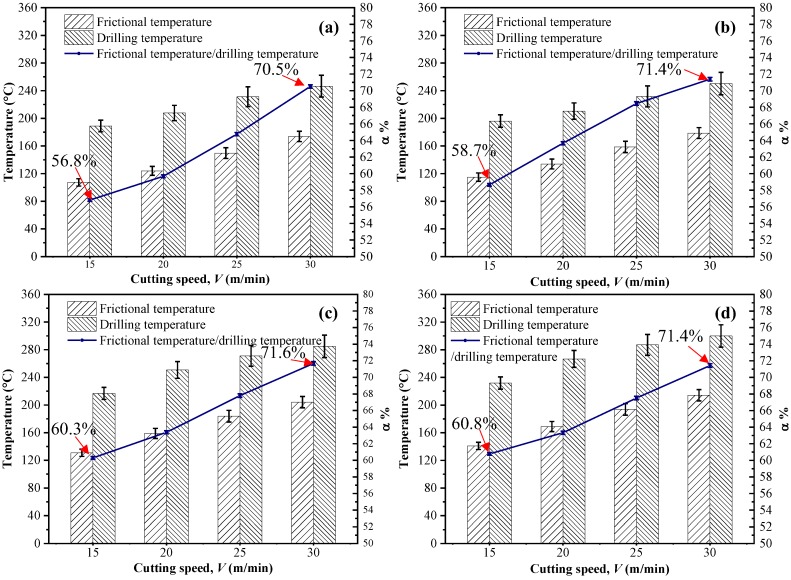
Temperature variations during the drilling and frictional tests under different cutting parameters: (**a**) *f* = 0.01 mm/rev, (**b**) *f* = 0.02 mm/rev, (**c**) *f* = 0.03 mm/rev and (**d**) *f* = 0.04 mm/rev.

Figure 9 shows the temperatures measured in both the drilling experiments and the frictional tests under different cutting conditions. The results indicate that the cutting speed has a positive impact on the heat generated during the drilling of CFRP composites. This is similar to the cases of cutting conventional metals in which the cutting temperature becomes higher when the speed is elevated. Additionally, the temperature changes marginally subjected to the varying feed rates, which is attributed to the fact that in drilling CFRPs, the dominant source of heat generation is the friction arising from the tool-work interaction zone that is more sensitive to the cutting velocity. Meanwhile, results of temperatures gained in the drilling and pin-on-disk tests show that the frictional temperature tends to account for a larger proportion of the overall drilling temperature when the cutting velocity increases. The highest proportion of 71.6% takes place under the conditions of *V* = 30 m/min and *f* = 0.03 mm/rev. This can be explained by the fact that the tool-work interface, which features the tribological interaction between the tool flank face and the machined composite surface, plays a vital role in the frictional heat generation, due to the elastic recovery effects resulting from the abrasive machined carbon fibers.

## 4. Conclusions

The present paper made an investigation on the tribo-drilling of high-strength CFRP composites by addressing the aspects of frictional heat and drilling temperatures. A particular focus was placed on the predictions of the frictional heat occurring at the flank tool-work interface and on the quantifying the contribution of the frictional temperature to the overall drilling temperature subjected to varying cutting conditions. According to the results obtained, the following key conclusions can be drawn.

- The interfacial friction coefficient shows a high sensitivity to the input drilling variables. Increasing the sliding velocity tends to reduce the magnitudes of both the *μ_app_* and *μ_adh_* due to the effects of thermal softening.
- The established analytical model can be used to predict the drilling temperatures of the tool-work interface under the condition of low feed rates. The experimental investigation confirms the presence of the contribution of the elastic recovery phenomenon to the interfacial friction coefficient, and the dominant source of heat generation when drilling CFRP composites is the friction taking place at the tool-work interaction zone, which contributes a percentage of around 71.6% to the overall drilling temperatures particularly under the cutting conditions of *V_c_* = 30 m/min and *f* = 0.03 mm/rev. To reduce the temperature of the flank tool-work surface, low cutting speeds and high feed rates, as well as the efficient geometry design of tools should be adopted.

## Figures and Tables

**Table 1:** Physical properties of the studied T800/X850 CFRP composite.

Material	Density (g/cm^3^)	Tensile Modulus (GPa)	Compressive Modulus (MPa)	Thermal Conductivity (W/(m⋅K))	Specific Heat Capacity J/(kg⋅K)
T800/X850	1.6	180	160	~15.10 × 10^−2^	0.18

**Table 2:** Cutting conditions used in the drilling of CFRP laminates.

Cutting speed, *V_c_* (m/min)	15, 20, 25 and 30
Feed rate, *f* (mm/rev)	0.01, 0.02, 0.03 and 0.04
Cutting Environment	Dry Conditions
